# An Unwonted Complication of Percutaneous Endoscopic Gastrostomy: A Case Report

**DOI:** 10.7759/cureus.3518

**Published:** 2018-10-29

**Authors:** Aniruthan Deivasigamani, Panneerselvam Vinodhini, Thirugnanam Nelson, TP Elamurugan, Sreenath GS

**Affiliations:** 1 Surgery, Jawaharlal Institute of Postgraduate Medical Education and Research (JIPMER), Puducherry, IND; 2 Surgery, Jawaharlal Institute of Postgraduate Medical Education and Research (JIPMER), Puducherry , IND

**Keywords:** buried bumper syndrome, percutaneous endoscopic gastrostomy, gastrostomy, peg, bbs

## Abstract

Percutaneous endoscopic gastrostomy (PEG) is a commonly used minimally invasive procedure to provide safe and durable access for long-term enteral nutrition in patients when oral feeds are not possible. The reported complications of PEG range from minor wound infections to life-threatening complications like hemorrhage and peritonitis. The buried bumper syndrome is one of the uncommon complications with a reported incidence of 0.3 to 2.4%. Though it is considered to be a late complication, the buried bumper syndrome has been reported as early as two weeks following the procedure. A thorough knowledge about this unusual complication is important for the prevention, early recognition and successful management of this condition to avoid interruption of enteral nutrition to the patient. Here we report a case of buried bumper syndrome developed at four weeks after placement of PEG tube.

## Introduction

Since its introduction in 1980, percutaneous endoscopic gastrostomy (PEG) has become a common procedure and is widely performed to provide access for enteral nutrition in patients when oral feeds are not possible. It is considered to be safe and easier than the open feeding procedures. The complications associated with this procedure range from minor wound infections to life-threatening complications like hemorrhage and peritonitis [1]. One of the uncommon complications of PEG is the ‘buried bumper syndrome’ with a reported incidence of 0.3 to 2.4% [2]. It is the migration of the inner bumper of PEG tube into or through the abdominal wall causing blockage to the feeds. It is usually considered to be a late complication and presents three to six months after the procedure. Early presentation of buried bumper syndrome is very rare and only a few cases have been reported in literature presenting as early as two weeks after PEG [3,4]. Various causes have been proposed for the buried bumper syndrome, like excess traction applied to the tube externally, type and rigidity of the PEG tube material, and obesity. Numerous techniques have been described in the literature for the removal of the migrated PEG tube and also to insert a new one for continuous enteral nutrition [2].

## Case presentation

A 25-year-old male was brought to our emergency department following a road traffic accident in July 2018. The patient was intubated in view of poor score on Glasgow Coma Scale (GCS). He was hemodynamically stable. On clinical examination, the patient had bilateral decreased air entry and positive chest compression. There were no abdominal signs. On focused assessment with sonography in trauma (FAST) there was minimal free fluid in the abdomen. Contrast-enhanced computed tomography (CECT) of thorax and abdomen showed bilateral hemopneumothorax and a grade III liver laceration. Initial non-contrast computed tomography (NCCT) of brain showed no intracranial injury but later the patient was declared to have a diffuse axonal injury and was shifted to critical care unit for monitoring. The liver laceration was managed conservatively since the patient was hemodynamically stable. Hemopneumothorax was managed with bilateral intercostal drains. The patient’s GCS was persistently poor. Initially, the patient was started on enteral feeds through nasogastric tube and later a PEG tube placement was planned for the purpose of continuing enteral feeds.

The procedure was performed while the patient was on endotracheal tube, and under intravenous (IV) sedation. A 20 Fr PEG tube was placed by the standard ‘Pull’ technique. Second look endoscopy confirmed the position of the internal bumper against the anterior wall of the stomach. Externally the tube was fixed and free flow of saline through the PEG was confirmed. There were no complications during the procedure and the patient was started on enteral feeds through the PEG tube on the same day. The patient was extubated after few days but he was continued on PEG feeds as his GCS was persistently poor.

**Figure 1:**
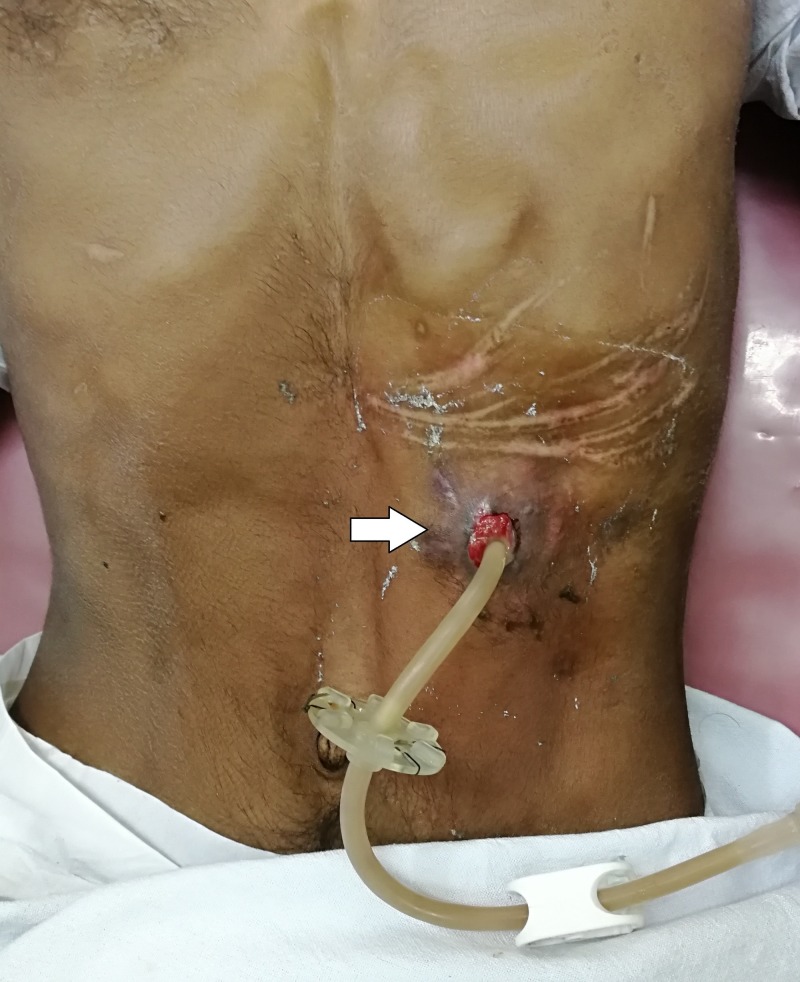
Percutaneous endoscopic gastrostomy tube insertion site. Showing a bulge with sprouting granulation tissue (arrow) at the percutaneous endoscopic gastrostomy insertion site.

**Figure 2:**
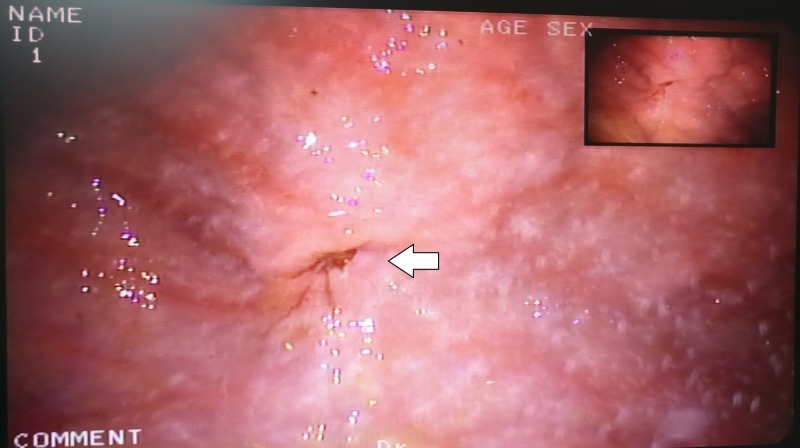
Endoscopic examination at four weeks showing a dimple (arrow) in the gastric mucosa suggestive of the tract through which the internal bumper had migrated outside.

**Figure 3:**
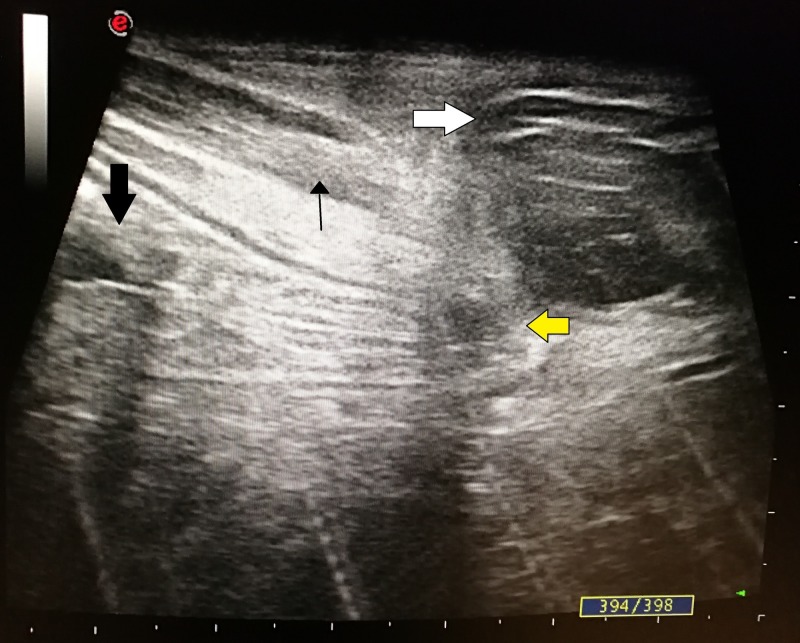
Ultrasonography (USG) image showing the internal bumper (white arrow) seen within the rectus abdominis muscle (thin black arrow). Thick black arrow showing the gastric wall, and yellow arrow showing the tract through which the internal bumper had migrated.

At four weeks after PEG there was peritubal leakage noted during feeds with resistance to the flow of feeds initially, which later progressed to complete the blockage. On examination, there was a slight bulge at the site of PEG tube insertion. There was granulation tissue visible sprouting through the tract externally (Figure 1). The patient had no signs of peritonitis and the abdomen was soft. On flushing the tube with saline, peritubal leakage was noted and there was resistance to flow. Endoscopic examination was performed to visualize the position of the internal bumper. On endoscopy, the internal bumper was not visualized. Only a small dimple was seen in the mucosa of the anterior wall of the stomach (Figure 2). The internal bumper appeared to have migrated through the tract and was entirely covered by the gastric mucosa with only a small dimple seen at the site of the tract. An ultrasound of the abdomen was performed which showed that the internal bumper was in the intramuscular plane of the rectus abdominis muscle (Figure 3).

**Figure 4:**
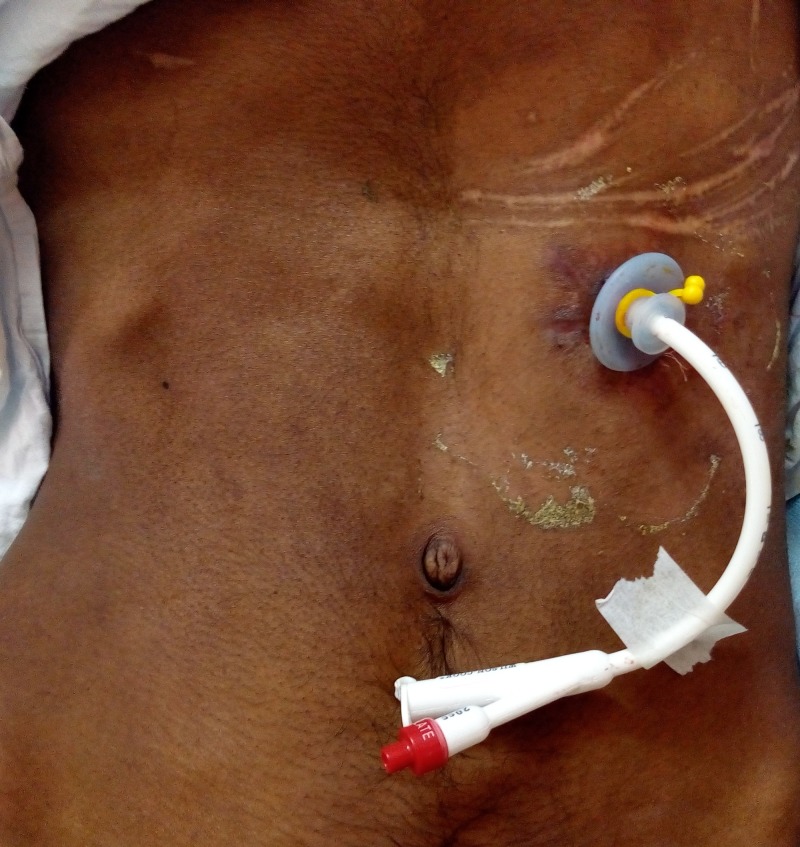
New balloon replacement gastrostomy tube in position.

As the patient’s GCS was still poor with no mature swallowing reflex, we planned to remove the old PEG tube and replace it with a new one for continued enteral feeding. The procedure was performed under general anaesthesia. Under fluoroscopic guidance, a guide wire was passed through the previous PEG tube from outside piercing the gastric wall. Then the old PEG was removed by gentle firm traction and the tract was dilated using dilator passed over the guide wire. The position of the dilator inside the stomach was confirmed by injecting a contrast dye under fluoroscopic visualization. Since the tract was well formed, a 20 Fr balloon replacement gastrostomy tube was inserted from outside and secured (Figure 4). Once again the position was confirmed by fluoroscopy and free flow of saline. The patient was started on PEG feeds later on the same day and had no complications. The patient was followed up for a month and was on continuous PEG feeds without any complications.

## Discussion

The first percutaneous endoscopic gastrostomy was reported in the literature by Gauderer et al. in 1980 [5]. Since then it has become a common and safe procedure to provide access for long-term enteral nutrition to patients when oral feeding cannot be initiated. Buried bumper syndrome is an uncommon complication of PEG tube placement and it is usually considered as a late complication [3]. The first case of migration of the inner bumper of PEG tube into or through the abdominal wall was reported in 1988 and later the name ‘buried bumper syndrome’ was coined.

The main underlying cause is attributed to be the close tight approximation of the internal bumper to the gastric wall, causing pressure necrosis and eventually leading to the migration of the internal bumper into the tract through the gastric wall and sometimes into the parietal wall or even into the subcutaneous plane. And the gastric mucosa grows over the buried bumper closing the tract completely. Undue excessive traction on the tube externally, rigid PEG tube, keeping a gauze under the external bumper, obesity etc. are all contributing factors for the buried bumper syndrome [6].

The most common and early presentation is the peritubal leakage of gastric feeds which progresses to increased resistance to the flow initially and later a complete blockage to the flow of feeds. Other presenting complaints include pain, discharge and rarely a swelling visible at the PEG site. At the site of PEG tube insertion there may be erythema, granulation tissue and sometimes the migrated internal bumper may be palpable if it is in the subcutaneous plane [1]. In this patient there was peritubal leak noted initially during feeds and there was increasing resistance to the flow of feeds. Later within few days, there was complete obstruction to the flow of feeds. There was a slight bulge at the PEG tube insertion site with granulation tissue sprouting through the tract.

Endoscopic examination usually shows an internal bumper partially or completely covered by gastric mucosa. In case the internal bumper is not at all visualized the tract may be seen as a dimple or a healed scar, as seen in our case. The position of the migrated internal bumper can be confirmed by ultrasonography (USG) or a computed tomography (CT) scan [2].

Various techniques have been described for the management of buried bumper syndrome ranging from the ‘cut and leave’ approach in case the PEG feeds is no longer required, to the removal of the migrated PEG tube and insertion of a new PEG tube [7-9]. In this case, we removed the buried PEG tube over a guide wire and inserted a balloon replacement gastrostomy tube under fluoroscopic guidance. This procedure did not require an endoscopic examination and since the tract was well formed, a replaceable gastrostomy tube was inserted from the outside and secured by inflating the inner balloon.

Buried bumper syndrome is a rare but a preventable complication of PEG. Avoidance of traction at the PEG fixation site, education to the staff and the patient relatives, and choosing a soft pliable PEG tube are all important in the prevention of this unusual complication. After two weeks of PEG insertion, unfastening the external bumper and inserting the PEG tube a few millimeters inside and rotating the tube 360 degrees regularly, also has been described to prevent migration of the internal bumper [2,10].

## Conclusions

The buried bumper syndrome is becoming one of the important problems following PEG and its incidence is anticipated to increase as the number of PEG procedures performed is increasing every day. A thorough knowledge about this condition is important to recognize the complication early in the course and to avoid interruption of continuous enteral feeds. As there are numerous techniques described for the safe removal and replacement of the PEG tube described in the literature, the appropriate management plan has to be chosen based on the patient factors, available materials and the experience of the surgeon.
